# Dual inhibition of glycolysis and glutaminolysis as a therapeutic strategy in the treatment of ovarian cancer

**DOI:** 10.18632/oncotarget.18854

**Published:** 2017-06-29

**Authors:** Li Sun, Yajie Yin, Leslie H. Clark, Wenchuan Sun, Stephanie A. Sullivan, Arthur-Quan Tran, Jianjun Han, Lu Zhang, Hui Guo, Esther Madugu, Tommy Pan, Amanda L. Jackson, Joshua Kilgore, Hannah M. Jones, Timothy P. Gilliam, Chunxiao Zhou, Victoria L. Bae-Jump

**Affiliations:** ^1^ Department of Gynecologic Oncology, Shandong Cancer Hospital Affiliated to Shandong University, Shandong Academy of Medical Sciences, Jinan, Shandong Province, People's Republic of China; ^2^ School of Medicine and Life Sciences, University of Jinan, Shandong Academy of Medical Sciences, Jinan, Shandong Province, People's Republic of China; ^3^ Division of Gynecologic Oncology, University of North Carolina at Chapel Hill, Chapel Hill, NC, USA; ^4^ Department of Surgical Oncology, Shandong Cancer Hospital Affiliated to Shandong University, Shandong Academy of Medical Sciences, Jinan, Shandong Province, People's Republic of China; ^5^ Division of Gynecologic Oncology, University of Cincinnati, Cincinnati, OH, USA; ^6^ Houston Methodist Gynecologic Oncology Associates, Houston, TX, USA; ^7^ Lineberger Comprehensive Cancer Center, University of North Carolina at Chapel Hill, Chapel Hill, NC, USA

**Keywords:** glycolysis, glutaminolysis, ovarian cancer, AMPK/mTOR

## Abstract

Cancer cell metabolism is required to support the biosynthetic demands of cell growth and cell division, and to maintain reduction oxidaton (redox) homeostasis. This study was designed to test the effects of glucose and glutamine on ovarian cancer cell growth and explore the inter-relationship between glycolysis and glutaminolysis. The SKOV3, IGROV-1 and Hey ovarian cancer cell lines were assayed for glucose, pyruvate and glutamine dependence by analyzing cytotoxicity, cell cycle progression, apoptosis and ATP production. As determined by MTT assay, glucose stimulated cell growth while the combination of glucose, glutamine and pyruvate resulted in the greatest stimulation of cell proliferation. Furthermore, 2-deoxy-glucose (2-DG) and 3-bromopyruvate (3-BP) induced apoptosis, caused G1 phase cell cycle arrest and reduced glycolytic activity. Moreover, 2-DG in combination with a low dose of aminooxyacetate (AOA) synergistically increased the sensitivity to 2-DG in the inhibition of cell growth in the ovarian cancer cell lines. These studies suggest that dual inhibition of glycolysis and glutaminolysis may be a promising therapeutic strategy for the treatment of ovarian cancer.

## INTRODUCTION

Ovarian cancer is the leading cause of death among gynecological malignancies and remains the 5th leading cause of cancer death among women in the United States [1]. Almost 75% patients with ovarian cancer are diagnosed with late stage disease. The initial treatment for advanced stage ovarian cancer frequently includes a surgical staging or debulking procedure followed by combination platinum and taxol adjuvant chemotherapy [2, 3]. Despite a high response rate to first-line chemotherapy, the majority of women with advanced stage ovarian cancer will relapse, with a median progression free survival (PFS) being 16 months after initial diagnosis. Further, 5 year survival is dismal at less than 40% [4, 5]. Therefore, new treatment strategies are urgently needed to improve outcomes in women with ovarian cancer.

The relationship between energy metabolism and tumorigenesis has been appreciated for several decades when Dr. Otto Warburg first described aerobic glycolysis as a metabolic hallmark of cancer metabolism [6]. Cancer cell metabolism is necessary to fuel the biosynthetic demands of cell growth, cell division and to maintain redox homeostasis. These changes in metabolism are, in fact, a direct result of the metabolic reprogramming of cells controlled by oncogenes and tumor suppressor genes [7, 8]. Regardless of the presence of adequate oxygen, cancer cells selectively utilize glycolysis over oxidative phosphorylation. Aerobic glycolysis in cancer cells utilizes glucose and glutamine as the primary carbon sources for ATP production and biosynthesis. Although cancer cells exhibit high rates of glycolysis, their mitochondrial oxidative phosphorylation remains intact and becomes progressively more dependent on glutamine metabolism to provide intermediates of the tricarboxylic acid (TCA) cycle to feed other biosynthetic pathways [7, 9].

Epidemiologic data show an increase risk of ovarian cancer in patients with type 2 diabetes [10]. Additionally, ovarian cancer patients with diabetes have been shown to have poorer survival [11]. Recent studies have confirmed that ovarian surface epithelial cells of mice representing early (benign), intermediate, and late (aggressive and invasive) stages of ovarian cancer display an increasingly glycolytic phenotype, suggesting that glycolysis is integral to the development and progression of ovarian cancer [12, 13]. Inhibition of glucose uptake or targeting the glycolytic pathway has shown promising anti-cancer effects in ovarian cancer cells and pre-clinical mouse models [14–16]. Quantitative metabolic parameters measured on FDG PET/CT at the time of the first relapse have significant predictive values for post-relapse survival in ovarian cancer [17]. In addition, we have recently demonstrated that restriction of glutamine or inhibition of glutaminase by compound 968 induces apoptosis and cell cycle arrest in ovarian cancer cells [18, 19]. Taken together, these data suggest that glycolysis and glutaminolysis are critically important in the pathogenesis of ovarian cancer. Thus, the aim of this study was to investigate the effects of glucose and glutamine on proliferation in ovarian cancer cells, and determine the potential of targeting glucose and glutamine metabolism as a promising therapeutic strategy for ovarian cancer.

## RESULTS

### Glucose is essential for cell survival

**Figure 1:**
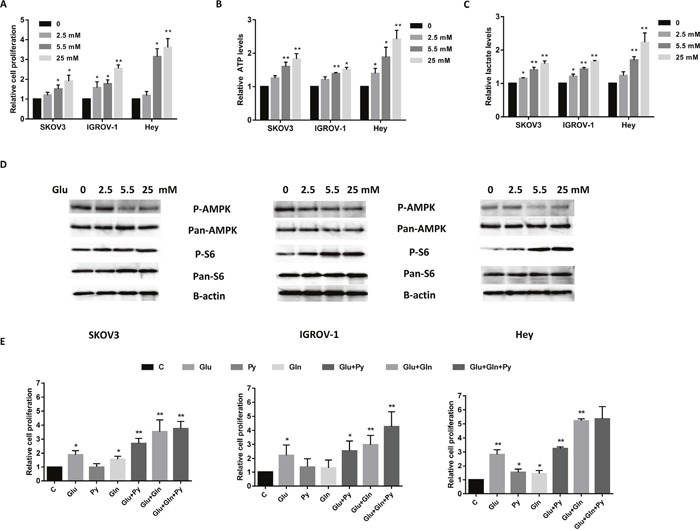
Glucose and glutamine are two major nutrients for cell proliferation in ovarian cancer cells The ovarian cancer cell lines SKOV3, IGROV-1 and Hey were treated in glucose free media supplemented with various concentrations of glucose (0, 2.5, 5.5, and 25 mM) for 48 hours. Cell proliferation was assessed by MTT assay. Glucose promoted cell proliferation **(A)**, increased ATP **(B)** and lactate production **(C)** in all three cell lines. The expression of phosphorylation of p-AMPK and p-S6 in SKOV3, IGROV-1 and Hey cells was detected by Western blotting after treatment with glucose for 24 hours **(D)**. The three cell lines were grown for 48 hours in media lacking glucose, glutamine or pyruvate supplemented with glucose (5.5 mM), glutamine (4.0 mM) or pyruvate (5.0 mM), respectively, or a combination of these as indicated **(E)**. The combination of glucose, glutamine and pyruvate caused maximal cell proliferation. The results are shown as the mean ± SEM of triplicate samples (*P<0.05, **p < 0.01) and are representative of three independent experiments.

We have previously shown that glucose is essential for the growth and survival of endometrial cancer cells [20]. To explore whether glucose modulates cell survival in ovarian cancer cells, we examined the effects of glucose alone on cell proliferation in three epithelial ovarian cancer cell lines. The SKOV3, IGROV-1 and Hey cells were cultured in their standard culture media with four concentrations of glucose (0, 2.5, 5.5 and 25 mM) for 72 hours. 5 mM glucose in the media corresponds to normal physiological levels in human blood (100 mg/dl), whereas 25 mM glucose is equivalent to a patient with severely uncontrolled diabetes [20]. Glucose effectively promoted cell proliferation in a dose dependent manner in all three cell lines (Figure 1A). To further examine how glucose affects energy flux, the cellular levels of ATP and lactate were measured in the ovarian cancer cells. Incubation in various glucose concentrations for 24 hours revealed that increasing glucose concentrations significantly increased cellular ATP level and lactate production (Figure 1B and 1C). These data suggest that SKOV3, IGROV-1 and Hey cells are glucose-dependent, with the Hey cells being the most sensitive to glucose stimulation.

AMP-activated protein kinase (AMPK) and the mammalian target of rapamycin (mTOR) are important protein kinases in maintaining cellular energy homeostasis and glucose metabolism in cancer cells [20, 21]. To investigate the mechanisms underlying the regulation of cell proliferation by glucose, we characterized the effect of glucose on the AMPK and mTOR/S6 signaling pathways. Glucose decreased phosphorylation of AMPK and increased phosphorylation of S6 in a dose-dependent manner in ovarian cancer cells within 24 hours after exposure (Figure 1D). These results indicate that the AMPK and mTOR/S6 pathways are involved in glucose metabolism in ovarian cancer.

Glucose, glutamine and pyruvate are the main nutrient sources used by cancer cells for biosynthesis, growth and survival [9]. To further validate the energy source of cell proliferation in ovarian cancer cells, the three cell lines were treated with the glucose (5.5 mM), glutamine (4.0 mM) or pyruvate (5.0 mM) alone or in combination in glucose/glutamine/pyruvate-free culture media for 48 hours. Cell death resulted in all three cell lines when cultured for 5 days under conditions of glucose starvation; cell death of these cell lines was continued to be induced even when their media was subsequently supplemented with glutamine and/or pyruvate (data not shown). Glucose alone can significantly maintain cell growth in all three ovarian cancer cell lines. In the absence of glucose in the culture media, either glutamine or pyruvate can temporarily maintain cell survival but for only 48 hours. All cell lines exhibited maximal cell proliferation in the media containing the mixture of glucose, glutamine and pyruvate (Figure 1E). Together, these results confirm that for these ovarian cancer cells, glycolysis is the main source of energy production, and glucose is the most critical nutrient for cell proliferation and survival.

### Glucose deprivation induces apoptosis in ovarian cancer cells

**Figure 2:**
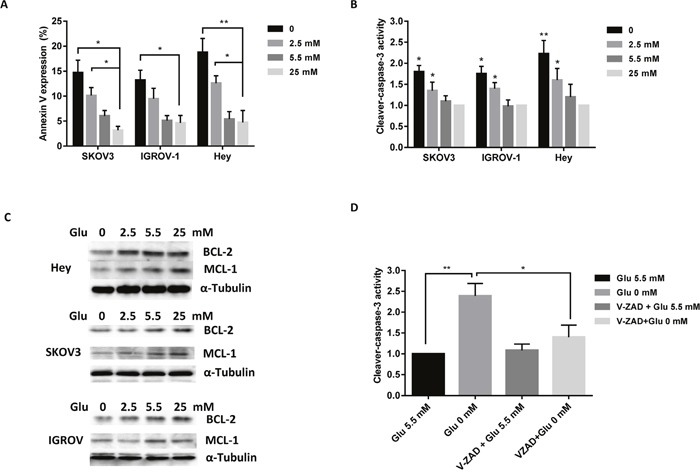
Depletion of glucose induces apoptosis in ovarian cancer cells The SKOV3, IGROV-1, and Hey cells were cultured with different concentrations of glucose (0, 2.5, 5.5, and 25 mM) for 14 hours. Glucose deprivation induced Annexin V expression **(A)** and increased cleaved caspase-3 activity **(B)**. Expression levels of Mcl-1 and Bcl-2 were analyzed by Western blot **(C)**. Hey cells were pre-treated using V-ZAD (20 mM) for 2 hours, and then treated with glucose (0, 5.5 mM). V-ZAD reduced cleaved caspase-3 activity induced by glucose deprivation **(D)**. Data are shown as the mean ± SEM of triplicate samples and are representative of three independent experiments.

To elucidate the mechanisms of glucose on cell growth, the effect of different concentrations of glucose on the induction of apoptosis was analyzed. SKOV3, IGROV-1 and Hey cells were treated with four concentrations of glucose in their standard media for 14 hours. The expression of Annexin V was increased distinctly under glucose deprivation compared to 25 mM glucose treatment (high glucose condition) in all three cell lines (Figure 2A). To further validate the effect of glucose on apoptosis pathways, ELISA assay analysis was used to detect the activity of cleaved caspase-3. Incubation of cells with glucose deprivation for 10 hours significantly increased cleaved caspase3 activity by approximately 80 to 120% in these three cell lines compared with the normal glucose groups (Figure 2B). Western blotting indicated that glucose deprivation decreased BCL-2 and MCL-1 protein expression in a dose dependent manner in all three cell lines (Figure 2C). To further analyze the role of glucose on the mitochondrial apoptosis pathway, we used pan-caspase inhibitor (Z-VAD-FMK) to block caspase activity in the Hey cells and determined whether caspase-3 activity was changed under glucose deprivation. Pre-treatment with Z-VAD-FMK for 2 hours resulted in complete blockage of glucose deprivation induced cleaved caspase-3 activity in the Hey cells (Figure 2D). These findings suggest that inducing mitochondrial apoptosis may be a major mechanism to inhibit cell proliferation in ovarian cancer cells under glucose deprivation or low glucose conditions.

### Glucose deprivation induces cell cycle G1 arrest in ovarian cancer cells

**Figure 3:**
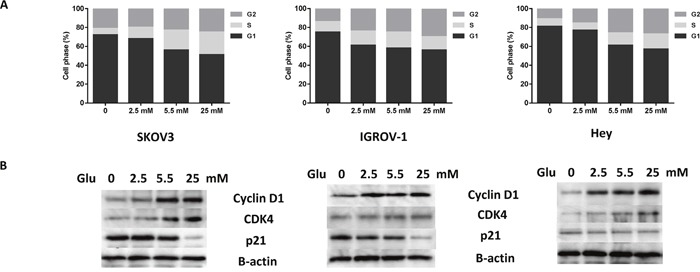
Glucose affects cell cycle progression in ovarian cancer cells The SKOV3, IGROV-1, and Hey cells were treated in glucose-free media supplemented with various concentrations of glucose (0, 2.5, 5.5, and 25 mM) for 36 hours. Cell cycle analysis was performed using Cellometer assay **(A)**. The effects of glucose on cyclin D1, CDK4 and p21 were examined by Western blot after exposure to glucose for 24 hours at the indicated concentrations **(B)**. Data are representative of three independent experiments.

To further confirm whether cell growth inhibition induced by glucose deprivation or low glucose, was related to cell cycle arrest, the cell cycle profile was analyzed by Cellometer after treating the SKOV3, IGROV-1 and Hey cells with four concentrations of glucose (0, 2.5, 5.5 and 25.0 mM) for 36 hours. The depletion of glucose increased the G1 population from 56% to 72% in SKOV3 cells, from 58% to 75% in IGROV-1 and from 61% to 81% in Hey cells when compared with normal glucose groups (5.5 mM). The S phase gradually increased with increasing concentrations of glucose in the three cell lines (Figure 3A). To further explore the effects of glucose on cell cycle checkpoints, the cells were cultured with the four concentrations of glucose for 24 hours. The results of western blot analysis showed that the expressions of both cyclin D1 and CDK4 increased, and p21 decreased with increasing glucose concentrations (Figure 3B). These results suggest that glucose promotes the passage of cells into S phase from G1 phase.

### Inhibition of glycolysis or glutaminolysis reduced cell proliferation in ovarian cancer cells

**Figure 4:**
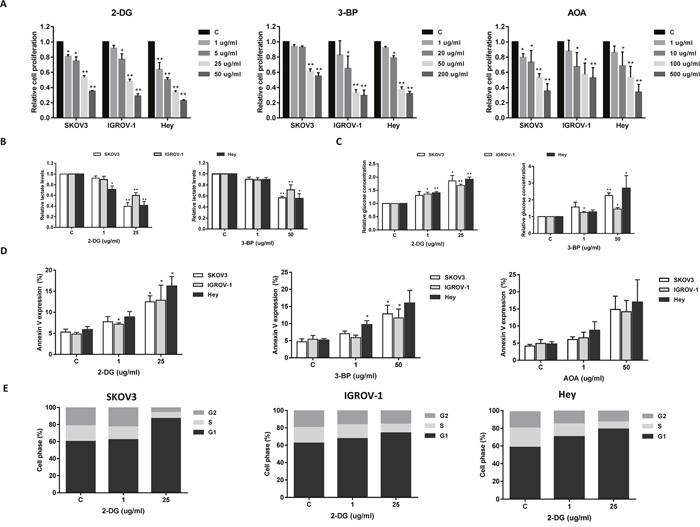
The effect of 2-DG, 3-BP and AOA on cell growth in ovarian cancer cells The SKOV3, IGROV-1, and Hey cells were treated with 2-DG, 3-BP and AOA for 48 hours as indicated. Cell proliferation was assessed by MTT assay. 2-DG, 3-BP and AOA reduced cell proliferation in a dose dependent manner **(A)**. 2-DG and 3-BP reduced lactate production and glucose consumptions in the media after 24 hours of treatment **(B** and **C)**. 2-DG, 3-BP and AOA increased the expression of Annexin V after 18 hours of treatment **(D)**. 2-DG induced cell cycle G1 arrest in all three cell lines **(E)**. Data are shown as the mean ± SEM of triplicate samples and are representative of three independent experiments.

To further analyze the effect of targeting glycolysis or glutaminolysis on cell growth in ovarian cancer cells, we treated the cells with two glycolysis inhibitors, 2-DG and 3-BP, and one glytaminolysis inhibitor, AOA. 2-DG is a glucose analogue that is unable to undergo glycolysis, and 3-BP targets both hexokinase-II (HK-II) and GAPDH in the glycolytic pathway [22]. AOA is a general inhibitor of pyridoxal phosphate-dependent enzymes including transaminases that are involved in amino acid metabolism [23]. Varying concentrations of 2-DG, 3-BP and AOA resulted in a dose dependent inhibition in cell growth in all three cell lines after 48 hours of treatment (Figure 4A). Consistent with their assumed antiglycolytic effects, 2-DG and 3-BP were able to increase the glucose concentration and reduce cellular lactate production in a dose dependent manner in all three cell lines, suggesting that 2-DG and 3-BP significantly reduced glucose consumption in the ovarian cancer cells (Figure 4B and 4C).

To evaluate the underlying mechanism of growth inhibition by 2-DG, 3-BP, and AOA, the effects of these inhibitors on cell apoptosis was analyzed using Annexin V assay. All three inhibitors significantly increased Annexin V expression in a dose dependent manner after 18 hours of treatment (Figure 4D). Analysis of cell cycle showed that only 2-DG caused a G1 phase arrest in the cells (Figure 4E) while 3-BP and AOA did not induce any changes in cell cycle even following incubation of the cells for 48 hours (data not shown). Together, these results indicate that targeting glycolytic or glutaminolytic pathways effectively inhibit cell growth in ovarian cancer cells.

### AOA synergizes with 2-DG to decrease cell proliferation in ovarian cancer cells

**Figure 5:**
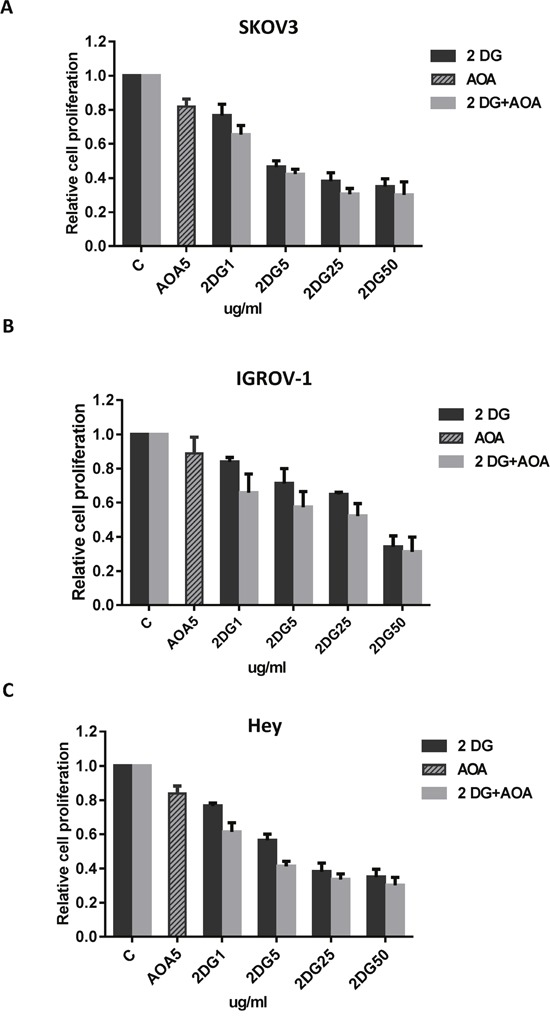
AOA increased sensitivity to 2-DG in inhibition of cell proliferation The SKOV3, IGROV-1, and Hey cells were cultured overnight and subsequently treated with AOA (5 ug/ml) alone and in combination with varying concentrations of 2-DG (1, 5, 25 and 50 ug/ml) for 72 hours. AOA synergistically enhanced anti-proliferative effect of 2-DG in ovarian cancer cells **(A-C)**. Data are shown as the mean ± SEM of triplicate samples and are representative of three independent experiments.

We have previously shown that inhibition of glutaminolysis results in a significant increase in glucose uptake in ovarian cancer cells. Thus, we hypothesize that AOA increases 2-DG uptake and sequentially sensitizes 2-DG in ovarian cancer cells. We treated the three ovarian cancer cell lines with AOA (5 ug/ml), or 2-DG (1, 5, 25 and 50 ug/ml) alone or a combination of the two for 72 hours. Cell proliferation was assessed by MTT assay. As expected, 2-DG alone resulted in a decrease in cell proliferation. Combining AOA with 2-DG at indicated concentrations, led to synergistic inhibitory effects in all three ovarian cancer cell lines (Figure 5A-5C, CI<1). These results suggested that inhibition of glutaminolysis results in increased sensitivity of ovarian cancer cells to glycolysis inhibitor 2-DG.

## DISCUSSION

In this study, we determined that glucose is the most important energy source in cell proliferation and investigated the potential role that glucose has in modulating cell growth in ovarian cancer cells. Our results show that the administration of glucose promotes cell growth though the inhibition of AMPK, the activation of mTOR/S6 pathways, the reduction of apoptosis and an increase in cell cycle S phase with corresponding increases in ATP and lactate production. In addition, targeting glycolysis with 2-DG and 3-BP, and targeting glutaminolysis with AOA effectively inhibit ovarian cancer cell proliferation. Moreover, AOA treatment resulted in significant synergy when combined with 2-DG to inhibit cell proliferation in ovarian cancer cells. These results support the concept for simultaneous targeting of glycolysis and glutaminolysis as a valuable strategy for the treatment of ovarian cancer.

Regardless of the availability of oxygen, cancer cells utilize high rates of glycolysis and lactate fermentation. This dependence on glucose utilization may reflect the fact that cancer cells are more sensitive to glucose stimulation than normal cells due to a higher consumption ratio of energy [20, 24]. The combination of glycolysis and glutaminolysis supports rapid proliferation of cancer cells through production of ATP and other biosynthetic precursors [25]. However, glycolysis alone yields enough energy to facilitate cancer cell growth despite mitochondrial defects [7]. Recent studies have confirmed that the response of cancer cells to glucose starvation is mainly dependent on the cell genetic and epigenetic background [26, 27]. The majority of cancer cells exhibit energy crisis under glucose deprivation viaactivation of cell death pathways that lead to apoptosis, cellular stress and cell cycle arrest [20, 28, 29]. Glucose deprivation induces G1 phase arrest and apoptosis in endometrial, breast, leukemia, prostate and lung cancer cells [20, 21, 27, 30–32]. However, in N-GlcNAc2-modified protein-producing renal carcinoma and bladder cancer cells, G2/M-phase arrest was seen following glucose deprivation [33, 34]. In this study, we evaluated the impact of glucose on cell cycle arrest and apoptosis in three ovarian cancer cell lines. Our results indicate that in these ovarian cancer cells, glucose deprivation causes inhibition of ovarian cancer cell growth through the induction of G1 phase cell cycle arrest and apoptosis as seen with augmented Annexin-V expression, along with decreased expression of CDK4, cyclin D1, MCL-1, BCL-2 and increased p21 expression. These results are consistent with our recent study in endometrial cancer [20] and suggest that the anti-proliferative effect induced by deprivation of glucose is associated with cell cycle arrest and apoptosis in ovarian cancer cells.

In the present study, we identified energy sources for cell proliferation in order to characterize the processes involved by targeting glycolysis and glutaminolysis in ovarian cancer. The growth of ovarian cancer cells relies primarily on glucose, although the tested cell lines exhibit different degrees of response to glucose stimulations. Either pyruvate or glutamine briefly keep the cells growing under conditions of glucose deprivation, and the combination of glucose, glutamine and pyruvate results in the greatest amount of cell proliferation. These findings suggest that the glycolytic pathway is the most important for energy generation and cell survival in ovarian cancer.

There are several approaches to disrupting glycolysis including targeting glucose transport, glycolytic processes, genes or pathways related to glycolytic metabolism [7, 9]. 2-DG is a reliable glycolysis inhibitors and a potential anti-tumorigenic agent [9, 35, 36]. Inhibition of glycolysis by 2-DG leads to decreased cellular ATP production, induction of cell cycle arrest, increased apoptosis and inhibition of cell proliferation in cancer cells [37–40]. Treatment of ovarian cancer cells with 2-DG resulted in a significant decrease in cell viability, increased caspase 3 activity, activation of AMPK and inhibition of AKT phosphorylation [41, 42]. The 3-BP mediated inhibition of glycolysis occurs mainly through targeting hexokinase-II (HK-II) and GAPD, which allows 3-BP to decrease the production of ATP, induce apoptosis, increase ROS and consequently cause growth inhibition in cancer cells including ovarian cancer [43]. The results of this study show that 2-DG and 3-BP have potent inhibitory effects along with induction of apoptosis on ovarian cancer cells that are comparable to glucose deprivation. Furthermore, increased extracellular glucose and decreased lactate production indicated that both 2-DG and 3-BP efficiently block glycolysis. Interestingly, in all three ovarian cancer cell lines, 2-DG treatment induced cell cycle arrest in G1 phase while 3-BP did not have this effect, suggesting that these two agents inhibit cell growth via different cytotoxic mechanisms.

Cancer cells utilize glucose and glutamine as primary carbon sources to feed mitochondrial intermediates for biosynthetic precursor. A recent study found that proliferating cells grown under hypoxic conditions rely almost exclusively on the reductive carboxylation of glutamine-derived α-ketoglutarate for de novo lipogenesis [44]. Our previous study in ovarian cancer cells found that either the deprivation of glutamine or the inhibition of glutaminolysis by compound 968 significantly reduced cell proliferation and induced apoptosis through AMPK/mTOR/S6 pathway [18, 19]. Therefore, it is possible that the combined inhibition of the reductive glutamine pathway together with inhibition of glycolytic flux would block multiple biosynthetic pathways and energy sources. This blockage may in turn lead to enhanced or synergistic inhibition of cancer cell viability. Indeed, the addition of metformin to 2-DG inhibited mitochondrial respiration and glycolysis in prostate cancer cells leading to a severe depletion in ATP [45]. The combination of metformin and 2-DG was much more inhibitory towards cancer cell proliferation than treatment with metformin or 2DG alone [45]. In the current study, we found that AOA inhibited cell growth in a dose dependent manner in ovarian cancer cells. Further, compared to each agent alone, combination of low dose AOA and serial doses of 2-DG lead to a strong synergistic cytotoxic effect on cell viability in all tested cancer cell lines. Given that the uncertainty of 2-DG as a single agent for anti-tumorigenic therapy, the combination of 2-DG and a glutaminolysis inhibitor may provide an innovative therapeutic strategy for ovarian cancer.

## MATERIALS AND METHODS

### Cell culture and reagents

The human ovarian cancer cell lines SKOV3, IGROV-1 and Hey were used. The Hey and SKOV3 cells were purchased from ATCC (American Type Culture Collection, USA). The IGROV-1 cell line was provided by Dr Jazaeri from the Department of Obstetrics and Gynecology, University of Virginia. The Hey and IGROV-1 cell lines were maintained in RPMI-1640 medium (cat # 21870-076, Gibco, USA) supplemented with 5% and 10% fetal bovine serum (FBS), respectively. The SKOV3 cell line was maintained in DMEM/(cat # 11966-025, Gibco, USA) medium supplemented with 10% FBS. For glucose studies, the cells were cultured in their standard maintenance medium containing 5% FBS and supplied with various concentrations of glucose (0, 2.5, 5.5, and 25.0 mM). All media was supplemented with 100 U/ml of penicillin and 100 ug/ml of streptomycin. All cells were cultured in a humidified 5% CO2 atmosphere at 37°C.

L-glutamine was purchased from Corning Cellgro (Manassas, VA). 3-(4,5-Dimethyl-2-thiazolyl)-2,5-diphenyl-2H-tetrazolium bromide (MTT), glucose solution, RNase A, 3-BP and AOA were purchased from Sigma-Aldrich (St. Louis, MO). The 2-DG and ATP assay kits were purchased from AAT Bioquest (Sunnyvale, CA). The Annexin V FITC kit was purchased from Biolegend (San Diego, CA). The anti-phosphorylated (phospho)-AMPK, anti-pan-AMPK, anti-phospho-S6, and anti-pan-S6 antibodies were all purchased from Cell Signaling (Beverly, MA). Enhanced chemiluminescence (ECL) detection reagents were purchased from GE Health Care (Piscataway, NJ). All other chemicals were purchased from Sigma.

### Cell proliferation assay

The SKOV3, IGROV-1 and Hey were seeded at 4000 cells/well in 96-well plates in their corresponding culture media. Twenty-four hours later, the cells were cultured in media containing different concentrations of glucose for 48 hours. Cell proliferation was measured by adding 5 ul MTT solution (5 mg/ml) per well for an additional 1 hour incubation. The MTT reaction was terminated by replacing the media with 100 ul DMSO. Viable cell densities were determined by measuring absorbance of metabolic conversion of the colorimetric dye at 570 nm (Tecan, Morrisville, NC). Each experiment was performed in triplicate and repeated three times.

### L-Lactate assay

Production of L-lactate was measured using the L-Lactate Assay Kit (Eton Bioscience, San Diego, CA). Briefly, the cells were treated with increasing concentrations of glucose, 2-DG and 3-BP for 24 hours. Next, 10 ul of the culture medium was transferred into a new 96-well plate; 40 ul of distilled water was subsequently added to each well. An additional 50 ul of glucose assay solution was added to each well, and the plates were incubated for 30 min at 37°C without CO2. Lactate levels were measured at a wavelength of 490 nm using a plate reader from Tecan. The experiments were performed in triplicate and repeated twice to assure consistency.

### Glucose assay

The concentrations of glucose in the media were detected by the Glucose Assay Kit from Eton Bioscience, following the manufacturer's instruction. After treatment of the cells with 2-DG and 3-BP for 24 hours, 10 ul of the culture medium and 40 ul distilled water were added into a new 96 well plates. An additional 50 ul of glucose assay solution was added to each well, and the plates were incubated for 15 min at 37°C without CO2. 50 μl of stop solution (0.5 M acetic acid) was added to each well immediately after incubation, followed by brief gentle agitation. Glucose levels were measured at 490 nm using a Tecan plate reader. Each experiment was performed in triplicate and repeated twice.

### ATP assay

The levels of cellular ATP was assessed using the Luminometric ATP Assay Kit. Briefly, cells were treated with glucose, 2-DG or 3-BP for 24 hours. 90 ul of the ATP reaction mix was added to each well and gently mixed. Samples were then incubated for 10-20 min, in the dark, at room temperature. The luminescence intensity was monitored with a plate reader from Tecan. ATP levels were normalized based on the viable cell counts measured by MTT assay. Each experiment was performed in triplicate and repeated twice.

### Cell cycle analysis

The effect of glucose, 2-DG and 3-BP on cell cycle progression was determined using Cellometer (Nexcelom, Lawrence, MA). Cells were plated at a density of 1.5 × 10^5^ cells/well in 6-well plates overnight and then treated with various concentrations of glucose, 2-DG and 3-BP for 36 hours. The cells were collected by 0.05% trypsin, washed with phosphate-buffered saline (PBS) solution, fixed in 90% methanol and then stored at −20°C until cell cycle analysis was performed. On the day of analysis, the cells were washed with PBS and centrifuged. Cells were then resuspended in 50 ul RNase A solution (250 ug/ml) with 10 mM EDTA, followed by incubation for 30 min at 37°C. After incubation, 50 μl propidium iodide (PI) staining solution (2 mg/ml PI, 0.1 mg/ml Azide, and 0.05% Triton X-100) was added to each tube and incubated for 10 min in the dark. The cells were then assessed by Cellometer. The results were analyzed using FCS4 express software (Molecular Devices, Sunnyvale, CA).

### Annexin V assay

The expression of Annixin V was detected using the Annexin-V FITC kit, according to the manufacturer's instructions. Briefly, 1.7 × 10^5^ cells/well were seeded into 6-well plates overnight, and then the cells were cultured in the media with various concentrations of glucose, 2-DG or 3-BP for 24 hours. The cells were collected by 0.25% trypsin without EDTA. After PBS washing, the cells were resuspended in 100 ul of Annexin-V and PI dual-stain solution (0.1 ug of Annexin-V FITC and 1 ug of PI) for 15 min in the dark and detected by Cellometer. The results were analyzed by the FCS4 express software. Each experiment was repeated at least twice for consistency of response.

### Cleaved caspase 3 assay

Cleaved caspase 3 was assessed with the Cleaved Caspase 3 Activity Assay kit. After treatment of the cells with different concentrations of glucose or V-ZAD in 96-well plates (6000 cells/well), 10 ul of caspase 3 assay loading buffer was added into each well, mixed gently and then the plates were incubated for 60 min at 37°C, 5% CO2. The fluorescence intensity was measured at an excitation wavelength of 350 nm and an emission wavelength of 450 nm using a plate reader from Tecan.

### Western blot analysis

Total protein was extracted from the ovarian cancer cells using RIPA buffer (Boston Bioproducts, Ashland, MA). Protein samples with equal loading (30 ug) were separated by 10-12% SDS-PAGE and transferred onto PVDF membranes. The membranes were blocked with 5% nonfat milk and then incubated with a 1: 000 dilution of primary antibodies overnight at 4°C. The membranes were washed and incubated with a secondary peroxidase-conjugated antibody for 1 hour at room temperature. The membranes were developed using an enhanced ECL at Alpha Innotech Imaging System (Protein Simple, Santa Clara, CA). After developing, the membranes were re-probed using antibody against α-tubulin or β-actin to confirm equal loading. The bands’ intensity were measured and normalized to α-tubulin. Each experiment was repeated at least twice for consistency of results.

### Statistical analysis

Data are expressed as mean ± SEM. Data were compared using two-tailed Student's t-test, and p < 0.05 was considered statistically significant.

Statistical analysis on synergy was analyzed by CalcuSyn (Biosoft, Cambridge, UK). The combination index (CI) of < 1 specifies a synergistic activity; whereas a CI value = 1 or a CI > 1 indicates additive and antagonistic effects, respectively.
